# Evaluation of Intracochlear Pressure and Fluid Distribution in 3D-Printed Artificial Cochlear Models and Human Petrous Bones †

**DOI:** 10.3390/brainsci15070771

**Published:** 2025-07-20

**Authors:** Rayoung Kim, Matthias Schürmann, Lars-Uwe Scholtz, Ingo Todt

**Affiliations:** Department of Otolaryngology, Head and Neck Surgery, Bielefeld University, Medical Faculty OWL, Clinic Bielefeld, Campus Mitte, Teutoburgerstr. 50, 33604 Bielefeld, Germany; matthias.schuermann@klinikumbielefeld.de (M.S.); lars-uwe.scholtz@klinikumbielefeld.de (L.-U.S.); todt@gmx.net (I.T.)

**Keywords:** second-hole technique, intracochlear substance application, intracochlear pressure, intracochlear dye distribution

## Abstract

**Introduction:** The important factor in applying substances for inner ear therapy is the atraumatic execution, as well as effective concentration uniformly distributed in all regions of the cochlea within a reasonable time frame. This study investigates whether an additional cochlear opening (“second-hole technique”) can improve fluid distribution and reduce intracochlear pressure during dye delivery into the cochlear models and human petrous bone. **Material and Methods:** Three experimental setups were used: an uncoiled scala tympani model, a full-scale 3D-printed cochlear model, and a human petrous bone. In all cases, 1% methylene blue-stained saline was infused using a cochlear catheter (MED-EL, Innsbruck, Austria) through the round window. Intracochlear pressure was measured via fiberoptic pressure sensors inserted through a burr hole (artificial cochlear models) or at the lateral semicircular canal (human petrous bone). A second hole was made on the helicotrema in the inner ear models or at the oval window of the human petrous bone to examine the effect of a second hole on intracochlear pressure and fluid distribution. Dye distribution and intracochlear pressure were measured in 3D artificial models at two flow rates (0.2 and 0.4 mL/h). The intracochlear pressure were measured in the human petrous bone at a fixed rate (0.4 mL/h). **Results:** The use of a second hole significantly improved dye distribution in 3D models at both flow rates (*p* < 0.05) and led to earlier saturation-level distribution. Intracochlear pressure remained significantly lower and more stable in models with a second hole (*p* < 0.05). In human petrous bones, pressure fluctuation was reduced by the second hole, though pressure still increased over time. **Conclusions:** Using a second-hole technique leads to a faster, uniform level of dye distribution throughout the cochlear models, as well as a lower intracochlear pressure, which can be assumed to be an essential factor for hearing preservation during dye application.

## 1. Introduction

According to the World Health Organization (WHO), more than 5% of the world’s population suffers from hearing loss. The most common form of hearing loss is known as sensorineural hearing loss (SNHL), which typically results from damage to the delicate mechanosensory structures in the inner ear [1].

At present, cochlear implants represent the most effective treatment option recommended for patients with severe to profound hearing loss. However, this technology has its limitations, resulting in restored hearing that falls short of natural hearing in several aspects, including poor tone discrimination and difficulty interpreting intonation [2].

Recent advances in cochlear pathophysiology have prompted growing interest in therapies aimed at preserving or restoring the native cochlear substrate, as opposed to bypassing it entirely, as cochlear implants do. These therapeutic strategies include pharmacologic agents (e.g., steroids) [3], small molecule drugs, neurotrophic factors such as brain-derived neurotrophic factor and neurotrophin-3 [4], as well as emerging gene and cell therapies that are expected to gain growing clinical relevance in the near future [5,6,7,8,9]. Notably, the success of gene therapy via injection of viral vectors has been demonstrated in multiple animal models [10,11,12], including embryonic models [13], as well as in human clinical studies [14,15].

These therapies typically require direct delivery to the inner ear to realize their full therapeutic potential. In a study by Pfannenstiel et al., adenoviral vector delivery to the cochlea via various routes in mice revealed that factors such as the delivery route and prior cochlear injury, rather than repeated dosing, are more critical in predisposing to additional hearing loss [16].

However, intracochlear drug delivery presents significant technical challenges. The cochlea is a tightly compartmentalized and fluid-filled structure, where even small changes in pressure can impact delicate structures and residual hearing. Many of these studies indicate that pressure changes in the cochlea caused by the intervention result in cochlear damage [17,18,19,20].

Greene et al. reported that intracochlear pressure transients during electrode insertion can reach levels comparable to those generated by high-intensity acoustic stimuli, potentially damaging the basilar membrane and hair cells [21]. Fernandez et al. provided experimental evidence in a mouse model that noise-induced synaptic damage (synaptopathy) can occur even without hair cell loss, underscoring the vulnerability of synapses to mechanical or pressure-related insult [22]. Elevated intracochlear pressure during drug administration may therefore cause direct mechanical trauma or microcirculatory disruption, potentially resulting in further injury to cochlear structures and hearing threshold shifts.

Therefore, there is a growing need to better understand intracochlear fluid dynamics during inner ear drug delivery in order to minimize structural damage and preserve residual hearing. Among the most critical factors are the intracochlear pressure changes and the distribution of the drug within the cochlea during injection.

To ensure safe and effective delivery, pressure fluctuations should be minimized, and a saturation-level distribution of the drug from the apex to the base of the cochlea should be considered a key objective.

In this study, we investigated whether an additional cochleotomy–referred to as the second-hole technique–could enable low and stable intracochlear pressure as well as saturation-level dye distribution during the dye injection through an opening analogous to the round window (referred to as ‘analog RW’) in the artificial cochlear models or actual round window (RW) in the human petrous bone. The applied methods were chosen to be transferable to clinical practice, including the timeframe and surgical approach. These findings provide a possible approach for inner ear therapy through drug injection, offering initial insights into the key factors that influence intracochlear pressure and the distribution of the drug within the inner ear compartments.

## 2. Materials and Methods

### 2.1. 3D-Printed Artificial Cochlear Models and Human Petrous Bone

**Figure 1:**
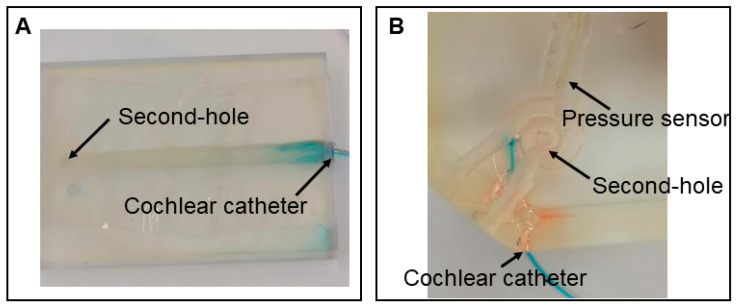
(**A**) The uncoiled scala tympani model, CC inserted to a depth of 5 mm into the analog RW. (**B**) The cochlear model, a pressure sensor, and CC inserted to a depth of 5 mm into the analog RW.

Two 3D-printed artificial cochlear models were used in the experiments. The first model was an uncoiled scala tympani model, designed based on a human cochlear computed tomography (CT) scan. It measured 3 cm in length with a volume of 100 mm^3^, which falls within the average anatomical range of a human cochlea [23]. The model consists of a cone-like hollow channel devoid of internal anatomical structures, such as the scala tympani, scala media, or scala vestibuli. It was fabricated using a 3D Systems MJP 2500 Plus printer (3D Systems, Rock Hill, SC, USA), with VisiJet M2R-Clear as the printing material. An additional drilled hole at the apex was included (Figure 1A).

The second model was a full-scale cochlear model with a total volume of 87 mm^3^. It replicated the overall shape and dimensions of a human cochlea but, like the first model, lacked any internal partitioning into anatomical structures such as the scala tympani, scala media, or scala vestibuli. This model was manufactured using a 3D Systems SLA 750 printer with Accura 60 Clear (3D Systems, Rock Hill, USA), an acrylic-based resin, as the printing material. Several additional burr channels were incorporated at the apex and the mid-turn of the cochlea (Figure 1B). In contrast to the uncoiled scala tympani model, this cochlear model features two additional drilled holes: one designated for pressure sensor insertion and the other serving as a secondary vent (“second-hole”) at 90° and 180° degree insertional depth at the lateral wall of the full scala (Figure 1B). Both models were printed with solid infill and no interface settings.

For the intracochlear pressure measurement in the human petrous bone, a canal-wall-up mastoidectomy was performed using an extended facial recess approach to expose critical middle and inner ear structures. This surgical access allowed direct visualization of the body and long process of the incus, the stapes, and the round window. To further optimize exposure of the round window niche, the facial canal was carefully opened, and the facial nerve was removed posterior to the oval and round windows.

### 2.2. Cochlear Catheter

**Figure 2:**
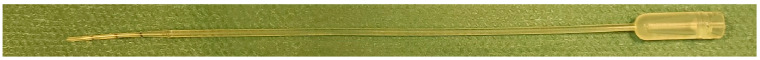
Cochlear catheter.

The CC utilized in our study is a silicone-based device specifically designed for the intracochlear delivery of substances (MED-EL, Innsbruck, Austria) [24]. The catheter features black markings at 5 mm, 10 mm, and 15 mm from the tip, facilitating precise depth measurements. The reservoir at the posterior end of the catheter is punctured with a needle to allow loading with the test substance (Figure 2). In our experiments, the dye-filled catheter was inserted to analog RW or RW. Several studies have already demonstrated that drug delivery via CC can be performed both atraumatically and effectively [25,26].

### 2.3. Pressure Sensor

The ICP was measured using a microoptical pressure sensor (FOP M200, FISO, Quebec, Canada) with a tip size of 0.2 mm. The tip of the pressure sensor is a hollow glass tube sealed on one end by a thin plastic film diaphragm coated with a reflective surface of evaporated gold. An optical fiber is located within the glass tube at a small distance (50–100 μm) from the diaphragm tip. The optical fiber is attached to an LED light source and a photodiode sensor. Light from the LED source reaches the sensor tip of the optical fiber. As the beam exits the fiber, it widens and is reflected by the gold-covered flexible diaphragm. The photodiode senses the reflected light. Small distance displacements of the diaphragm modulate the intensity of reflected light. The sensor is connected to a module that is linked to a computer. Intracochlear pressure was recorded using Evolution software (Version 2.2.0.0, FISO, Quebec, QC, Canada). The sensor operated at a time resolution of 300 Hz, while its measurement accuracy was independently specified as ±3% by the manufacturer.

### 2.4. Experimental Setup and Procedure

Experiments were conducted using three different specimen types: a 3D-printed uncoiled cochlear model, a standard cochlear model, and a human petrous bone. In all setups, 1% methylene blue-stained saline (0.9%, Merck KGaA, Darmstadt, Germany) was used to evaluate fluid distribution and intracochlear pressure (ICP) during catheter-based infusion.

#### 2.4.1. Specimen Preparation and Catheter Placement

All cochlear models were pre-filled with 0.9% saline solution. To prevent fluid leakage, all openings—including drilled burr holes for the pressure sensor and second hole, as well as the base aperture—were sealed with adhesive tape. In the cochlear models, an analog round window (RW) was punctured using a 0.4 mm perforator (Karl Storz, Tuttlingen, Germany), whereas in the human petrous bone, the anatomical RW was accessed in the same manner. A dye-filled cochlear catheter (CC; MED-EL, Innsbruck, Austria) was then inserted through the RW.

In the uncoiled model, the CC was inserted up to the first black marking at 5 mm. In the standard cochlear model and the human petrous bone, the catheter was advanced further, up to 8 mm.

To measure ICP, a fiberoptic pressure sensor (FOP M200, FISO, Québec, QC, Canada) was inserted via an additional burr hole in the cochlear model or through a puncture in the lateral semicircular canal (LSC) in the petrous bone.

#### 2.4.2. Sealing and Leak Prevention

To ensure leak-tight conditions at the catheter and sensor insertion sites, fibrin glue (Tisseel, Baxter International, Deerfield, FL, USA) was applied following placement of both the CC and the pressure sensor. This sealing step was performed in all experimental conditions, including both cochlear models and the human petrous bone. Approximately 1 mL of fibrin glue was applied per application site. In the case of the petrous bone, additional sealing using autologous connective tissue was performed at the catheter entry point. A curing time of at least two minutes was allowed after glue application before beginning measurements.

#### 2.4.3. Second-Hole Creation

**Figure 3:**
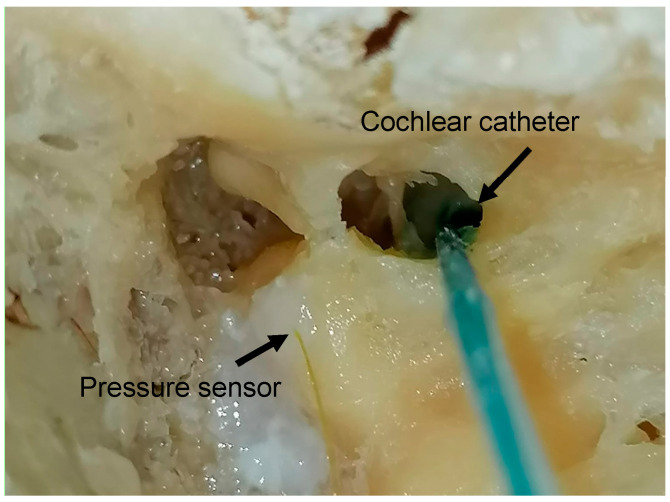
The experiment was conducted using human cadaveric petrous bones. Pressure sensors were inserted into the LSC and the cochlear canal near the RW, and were sealed in place using fibrin glue to prevent fluid leakage.

To investigate the effect of an additional fluid outlet, a second hole was created in all specimen types. In the cochlear models, this hole was consistently placed at the helicotrema using a 0.4 mm perforator. In the human petrous bone, the second-hole was created in the stapes footplate, also using a 0.4 mm perforator (Figure 3). This modification was intended to simulate pressure relief mechanisms and assess their influence on fluid distribution and ICP.

#### 2.4.4. Dye Injection and Flow Control

Dye delivery was performed using a continuous-infusion syringe pump (Fresenius, Bad Homburg, Germany) to maintain a steady flow. In the cochlear models, dye was infused at flow rates of 2 mL/h and 4 mL/h. In the human petrous bone, a flow rate of 4 mL/h was used. The catheter was held in a fixed position during all experiments to minimize positional variability. Each infusion lasted for 15 min.

#### 2.4.5. Measurement and Documentation

Dye distribution was documented photographically at predefined time points: 0, 210, 270, 390, 450, 510, 630, 750, and 900 s after the start of infusion.

In the uncoiled model, only dye distribution was analyzed. In the standard cochlear model, both dye distribution and ICP were assessed. In the human petrous bone, only ICP was measured. Pressure data were continuously recorded throughout the 15 min infusion and evaluated at the same time intervals as the photographic documentation.

### 2.5. Analysis

**Figure 4:**
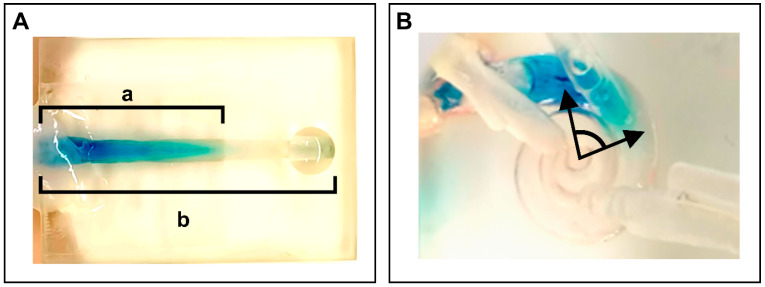
Measurement approaches in the two models. (**A**) Measurement of dye distribution in the uncoiled scala tympani model as a ratio (a/b). (**B**) Measurement of dye distribution in the cochlear model at an angle.

The distribution ratio in our uncoiled scala tympani model was calculated by dividing the length of the scala tympani reached by dye by the total length of the model’s scala tympani (a/b), as shown in Figure 4A. For the cochlear model, the distribution was determined by measuring the distribution angle reached, as illustrated in Figure 4B. The angular measurement was calculated by measuring the angle between two lines: one originating from the center of the modiolus to the tip of the inserted CC, and the other from the modiolus center to the furthest point of visible dye distribution. The modiolus served as the central reference point for all angular measurements. The intracochlear pressure was continuously monitored and recorded using Evolution software (FISO Technologies Inc., Québec, QC, Canada).

All statistical analyses were performed using GraphPad Prism (version 8.0.2). Data were expressed as mean values ± standard deviation (SD). Comparisons between groups (e.g., with vs. without second hole at different flow rates) were conducted using paired Student’s *t*-tests under the assumption of equal variances. A significance threshold of *p* < 0.05 was applied throughout. Each experimental condition was repeated at least three times (*n* ≥ 3), and average values were used for comparison. For distribution analyses, saturation-level thresholds were defined based on the point at which no further dye spread was observed.

## 3. Results

### 3.1. Dye Distribution With or Without a Second Hole in Models

**Figure 5:**
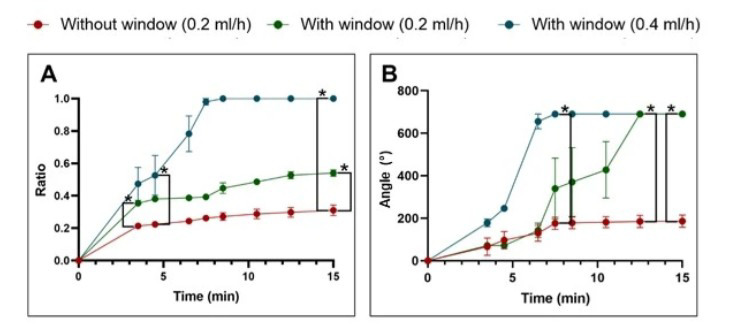
Measurements of the distribution of test liquid in artificial models with and without a second hole (**A**) in the uncoiled scala tympani model and (**B**) in the cochlear model. The perfusion at a high flow rate of 0.4 mL/h with the second hole (blue) resulted in the complete distribution of the test solution throughout the model within approximately 8.5 min. In contrast, in the absence of a second hole (red), intracochlear perfusion was limited, with distribution reaching less than 30% in both of the models (* = *p* < 0.05).

Dye application at a rate of 0.2 mL/h, with an additional second hole, resulted in significantly improved perfusion compared to application without a second hole (*n* = 5). However, complete distribution of the test fluid in the uncoiled scala tympani model was not achieved under initial conditions (distribution ratio: 0.31 vs. 0.54). At a higher perfusion rate of 0.4 mL/h and with the presence of a second hole (*n* = 3), a saturation-level distribution of the test fluid was successfully achieved, with statistical significance observed as early as 8 min after the start of the injection (Figure 5A).

In the cochlear model, the inclusion of a second-hole resulted in a complete and significantly improved distribution of the test fluid across all tested perfusion rates (*n* = 4). However, the time required to achieve complete perfusion varied considerably depending on the flow rate: 8.5 min at 0.4 mL/h and 12.5 min at 0.2 mL/h. In contrast, without a second hole (*n* = 4), fluid distribution was markedly limited, with less than 30% of the cochlear volume being perfused (Figure 5B).

### 3.2. Intracochlear Pressure in the Cochlear Model

**Figure 6:**
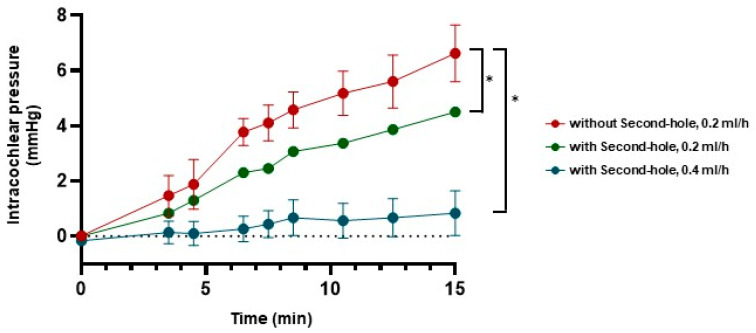
The pressure changes in the cochlear model during perfusion. Intracochlear pressure during perfusion at a rate of 0.4 mL/h with the second hole (blue) was more stable and lower compared to the pressure observed at a perfusion rate of 0.2 mL/h (red: without second hole, green: with second hole) (* = *p* < 0.05).

The pressure changes in the cochlear models were measured continuously at specific time points (Figure 6). Each experimental condition was tested at least three times. In models equipped with a second hole, intracochlear pressure remained significantly lower and more stable compared to models without a second hole. With a second hole, the pressure remained nearly constant at approximately zero mmHg, whereas in models lacking a second hole, the intracochlear pressure increased progressively over time.

### 3.3. Intracochlear Pressure in the Human Petrous Bone

**Figure 7:**
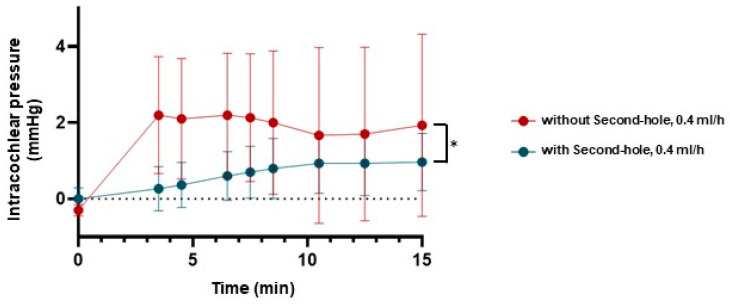
Measurements of the intracochlear pressure in the human petrous bone. Intracochlear pressure in the human petrous bone during perfusion at a rate of 0.4 mL/h with the second hole (blue) was more stable and lower compared to the pressure observed at a perfusion rate of 0.4 mL/h without the second hole (red) (* = *p* < 0.05).

Intracochlear pressure increased continuously at a perfusion rate of 0.4 mL/h, both with and without a second hole (*n* = 3). However, the fluctuation range was significantly larger in models without a second hole compared to those with a second hole (2.20 mmHg vs. 1.0 mmHg) (Figure 7).

## 4. Discussion

This study aimed to explore the impact of a second hole in the cochlea on the distribution of dye and intracochlear pressure dynamics. Using both uncoiled scala tympani and cochlear models, as well as human petrous bone samples, we evaluated how the presence or absence of a second hole affects fluid distribution and intracochlear pressure under different perfusion conditions. The results presented here provide valuable insights into the design of cochlear infusion systems and potential implications for clinical treatments, such as drug delivery or cochlear implantation.

### 4.1. Dye Distribution and the Role of the Second Hole

One of the most significant findings of this study was the substantial improvement in dye distribution in both the uncoiled scala tympani and cochlear models when a second hole was added. This finding supports the hypothesis that additional access points, such as a second hole at the apex of cochlear models, promote more uniform fluid dispersion. The uncoiled cochlear model, which lacks anatomical complexity, showed enhanced distribution with the second hole, with 50% of the model at a perfusion rate of 0.2 mL/h. A complete distribution was achieved at higher perfusion rates (0.4 mL/h) within 8 min. This suggests that the second hole facilitates the even spread of the fluid across the model’s length, minimizing stagnation in some areas of the cochlea.

In contrast, the uncoiled cochlear model lacking a second hole exhibited poor distribution, with less than 30% of the enclosed volume showing visible dye presence at a perfusion rate of 0.2 mL/h. This observation suggest that the absence of a second outlet may lead to insufficient perfusion and inefficient dye spread.

In the standard cochlear model, the second hole consistently resulted in complete perfusion across all perfusion rates, further emphasizing the importance of the second hole for effective cochlear drug delivery. Notably, the time required for full distribution was inversely related to perfusion rate, with a higher rate achieving complete distribution in less time. This finding aligns with established principles of fluid dynamics, which state that higher pressures and flow rates reduce the time required to reach equilibrium.

In numerous studies investigating pharmacokinetics within the inner ear after intracochlear substance injection, various locations have been targeted, including the RW [26,27,28], the posterior semicircular canal (PSCC) [29], and the cochlear apex [30].

Both fluid application and subsequent perilymph sampling via the RW have consistently demonstrated a basal-to-apical concentration gradient of the administered markers [27]. Yildiz et al. observed an increase in perilymph concentrations over time following RW drug application via CC, as evidenced by apical sampling at 2, 6, and 24 h post-application [26]. However, these sampling intervals are not clinically feasible due to their extended duration. Salt et al. reported that drug injection at the cochlear apex can lead to a more uniform distribution of markers along the cochlear length. Nevertheless, this finding was based on experiments in guinea pigs, which possess a scala tympani volume of approximately 4.7 µL [30]. In contrast, achieving an adequate drug concentration for clinical effect across the entire human cochlea appears to be more challenging, especially given that passive diffusion alone is unlikely to be sufficient. This is underscored by the considerably larger total inner ear volume in humans (~191.1 µL) compared to that in guinea pigs (~15.94 µL) [31].

Talaei et al. demonstrated a uniform dye distribution following intracochlear injection via the PSCC in adult mouse models [29]. However, in all of the studies as mentioned above, the substance application was conducted in a closed system (i.e., without a secondary vent hole). Importantly, these studies did not address the issue of intracochlear pressure, which plays a critical role in preserving residual hearing.

Our second-hole technique is particularly relevant for the design of cochlear drug delivery systems, where uniform distribution is critical to ensure the efficient delivery of therapeutic substances across the cochlea.

### 4.2. Intracochlear Pressure Dynamics

The intracochlear pressure measurements provided additional insights into the effects of a second hole on cochlear dynamics during dye infusion. Numerous studies have proposed that increased intracochlear pressure may affect residual hearing and recommend decreasing and controlling intracochlear pressure changes [19,21,32,33].

In the cochlear models in our study, the presence of a second hole significantly stabilized intracochlear pressure. Specifically, pressure change remained near zero mmHg when a second hole was included, whereas models without a second hole exhibited a continuous rise in intracochlear pressure over time. This suggests that the second hole alleviates the buildup of pressure within the cochlea, potentially reducing the risk to the intracochlear structures. When a second hole was created, intracochlear pressure remained consistently lower during dye injection at the higher infusion rate of 0.4 mL/h compared to 0.2 mL/h. A possible explanation is that, at higher flow rates, increased fluid leakage through the second hole may facilitate more effective pressure relief than at lower rates.

Interestingly, when examining human petrous bone samples, the results showed that intracochlear pressure continued to increase at a perfusion rate of 0.4 mL/h, regardless of the presence of the second hole. However, the fluctuations in pressure were less pronounced with a second hole, indicating a stabilizing effect of the second hole on intracochlear pressure dynamics.

Continuous elevation of intracochlear pressure in the absence of a secondary vent (second hole) may impose harmful mechanical stress on delicate cochlear structures. However, creating an additional cochleostomy to serve as a second-hole also carries the potential risk of residual hearing loss. Carvalho et al. examined auditory brainstem response (ABR) thresholds following intracochlear injection via cochleostomy in guinea pigs. They reported that a properly performed cochleostomy did not result in persistent cochlear dysfunction [34]. Nevertheless, the available studies have not sufficiently investigated the long-term impact of cochleostomy on cochlear function. Therefore, future research should rigorously assess and compare the potential risks and benefits of creating a second cochleostomy against the risk of pressure-induced intracochlear damage in a closed system.

### 4.3. Implications for Clinical Applications

The findings of this study hold several important implications for clinical applications involving cochlear drug delivery or implants. First, the use of a second hole in the cochlea may improve the efficacy and safety of cochlear drug delivery systems by facilitating more uniform distribution and maintaining pressure stability during infusion. In clinical practice, this could translate to more effective treatments for hearing loss or cochlear diseases, where consistent drug distribution is crucial.

Elevated intracochlear pressure during the implantation process can lead to complications such as membrane rupture or displacement of cochlear components. Therefore, creating a second hole during intracochlear drug application may reduce the risk of pressure-related complications and improve the overall success of the procedure.

### 4.4. Limitations and Future Research

Although this study provides valuable insights into cochlear dye distribution and pressure dynamics, several limitations must be addressed in future research. The artificial cochlear models, while useful for controlled experimentation, do not perfectly replicate the full anatomical and physiological complexity of the human cochlea. For example, the absence of separate anatomical structures, such as the scala tympani, scala vestibuli, and scala media, in the models may influence the flow of fluids in ways that differ from those in the human cochlea. Furthermore, the use of human petrous bone models is limited by the availability of viable samples.

The conclusions regarding dye distribution are based solely on visual assessment, and as such, the interpretation is qualitative in nature. To validate our findings and establish a more objective basis for evaluating flow dynamics within the model, quantitative measurements would be necessary.

Additionally, the study primarily focused on perfusion dynamics at fixed rates with or without a second hole. The presence of a second hole significantly improved both dye distribution and pressure stabilization. In addition, an increased flow rate had a substantial effect, but this was evident only when a second hole was present. Due to the absence of a control at high flow rate (0.4 mL/h) without a second hole, the independent effects of each variable cannot be fully separated. Future studies could investigate the effects of different infusion substances, including those with varying viscosities or biological properties, on fluid distribution and pressure in the cochlea.

## 5. Conclusions

In conclusion, the inclusion of a second hole in cochlear models significantly improves both dye distribution and intracochlear pressure stability during perfusion. These findings suggest that the second hole may be crucial for optimizing cochlear drug delivery systems, thereby enhancing the safety and efficacy of cochlear treatments. Future studies should explore the clinical feasibility of incorporating a second hole in cochlear implantation and drug delivery procedures to refine these approaches for patient care further.

## Data Availability

The data presented in this study are available on request from the corresponding author in accordance with institutional data sharing policies.

## References

[1] World Health Organization (2025). Deafness and Hearing Loss.

[2] Fletcher M.D., Thini N., Perry S.W. (2020). Enhanced pitch discrimination for cochlear implant users with a new haptic neuroprosthetic. Sci. Rep..

[3] Malkoc G., Dalgic A., Koc M., Kandogan T., Korkmaz S., Ceylan M., Inan S., Olgun L. (2014). Histopathological and audiological effects of mechanical trauma associated with the placement of an intracochlear electrode, and the benefit of corticosteroid infusion: Prospective animal study. J. Laryngol. Otol..

[4] Lye J., Delaney D.S., Leith F.K., Sardesai V.S., McLenachan S., Chen F.K., Atlas M.D., Wong E.Y. (2023). Recent therapeutic progress and future perspectives for the treatment of hearing loss. Biomedicines.

[5] Lin Q., Guo Q., Zhu M., Zhang J., Chen B., Wu T., Jiang W., Tang W. (2022). Application of nanomedicine in inner ear diseases. Front. Bioeng. Biotechnol..

[6] Roy S., Johnston A.H., Newman T.A., Glueckert R., Dudas J., Bitsche M., Corbacella E., Rieger G., Martini A., Schrott-Fischer A. (2010). Cell-specific targeting in the mouse inner ear using nanoparticles conjugated with a neurotrophin-derived peptide ligand: Potential tool for drug delivery. Int. J. Pharm..

[7] Saidia A.R., Ruel J., Bahloul A., Chaix B., Venail F., Wang J. (2023). Current advances in gene therapies of genetic auditory neuropathy Spectrum disorder. J. Clin. Med..

[8] Warnecke A., Prenzler N., Harre J., Köhl U., Gärtner L., Lenarz T., Laner-Plamberger S., Wietzorrek G., Staecker H., Lassacher T. (2021). First-in-human intracochlear application of human stromal cell-derived extracellular vesicles. J. Extracell. Vesicles.

[9] Warnecke A., Staecker H., Rohde E., Gimona M., Giesemann A., Szczepek A.J., Di Stadio A., Hochmair I., Lenarz T. (2022). Extracellular Vesicles in Inner Ear Therapies—Pathophysiological, Manufacturing, and Clinical Considerations. J. Clin. Med..

[10] Andres-Mateos E., Landegger L.D., Unzu C., Phillips J., Lin B.M., Dewyer N.A., Sanmiguel J., Nicolaou F., Valero M.D., Bourdeu K.I. (2022). Choice of vector and surgical approach enables efficient cochlear gene transfer in nonhuman primate. Nat. Commun..

[11] Mathiesen B.K., Miyakoshi L.M., Cederroth C.R., Tserga E., Versteegh C., Bork P.A., Hauglund N.L., Gomolka R.S., Mori Y., Edvall N.K. (2023). Delivery of gene therapy through a cerebrospinal fluid conduit to rescue hearing in adult mice. Sci. Transl. Med..

[12] Praetorius M., Knipper M., Schick B., Tan J., Limberger A., Carnicero E., Alonso M.T., Schimmang T. (2002). A novel vestibular approach for gene transfer into the inner ear. Audiol. Neurotol..

[13] Barbosa Spinola C.M., Boutet de Monvel J., Safieddine S., Lahlou G., Etournay R. (2024). In utero adeno-associated virus (AAV)-mediated gene delivery targeting sensory and supporting cells in the embryonic mouse inner ear. PLoS ONE.

[14] Lv J., Wang H., Cheng X., Chen Y., Wang D., Zhang L., Cao Q., Tang H., Hu S., Gao K. (2024). AAV1-hOTOF gene therapy for autosomal recessive deafness 9: A single-arm trial. Lancet.

[15] Qi J., Tan F., Zhang L., Lu L., Zhang S., Zhai Y., Lu Y., Qian X., Dong W., Zhou Y. (2024). AAV-Mediated Gene Therapy Restores Hearing in Patients with DFNB9 Deafness. Adv. Sci..

[16] Pfannenstiel S.C., Praetorius M., Brough D.E., Staecker H. (2020). Hearing preservation following repeated Adenovector delivery. Anat. Rec..

[17] Allen G.W. (1983). Clinical implications of experiments on alteration of the labyrinthine fluid pressures. Otolaryngol. Clin. N. Am..

[18] Böhmer A. (1993). Hydrostatic pressure in the inner ear fluid compartments and its effects on inner ear function. Acta Oto-Laryngol..

[19] Hartl R.M.B., Mattingly J.K., Greene N.T., Farrell N.F., Gubbels S.P., Tollin D.J. (2017). Drill-induced cochlear injury during otologic surgery: Intracochlear pressure evidence of acoustic trauma. Otol. Neurotol..

[20] Mittmann P., Ernst A., Todt I. (2014). Intracochlear pressure changes due to round window opening: A model experiment. Sci. World J..

[21] Greene N.T., Mattingly J.K., Hartl R.M.B., Tollin D.J., Cass S.P. (2016). Intracochlear pressure transients during cochlear implant electrode insertion. Otol. Neurotol..

[22] Fernandez K.A., Guo D., Micucci S., De Gruttola V., Liberman M.C., Kujawa S.G. (2020). Noise-induced cochlear synaptopathy with and without sensory cell loss. NeuroSci..

[23] Kirk E.C., Gosselin-Ildari A.D. (2009). Cochlear labyrinth volume and hearing abilities in primates. Anat. Rec. Adv. Integr. Anat. Evol. Biol. Adv. Integr. Anat. Evol. Biol..

[24] Dhanasingh A., Hochmair I. (2021). Drug delivery in cochlear implantation. Acta Oto-Laryngol..

[25] Prenzler N.K., Salcher R., Lenarz T., Gaertner L., Warnecke A. (2020). Dose-dependent transient decrease of impedances by deep intracochlear injection of triamcinolone with a cochlear catheter prior to cochlear implantation–1 year data. Front. Neurol..

[26] Yildiz E., Gadenstaetter A.J., Gerlitz M., Landegger L.D., Liepins R., Nieratschker M., Glueckert R., Staecker H., Honeder C., Arnoldner C. (2023). Investigation of inner ear drug delivery with a cochlear catheter in piglets as a representative model for human cochlear pharmacokinetics. Front. Pharmacol..

[27] Laurell G., Teixeira M., Sterkers O., Bagger-Sjöbäck D., Eksborg S., Lidman O., Ferrary E. (2002). Local administration of antioxidants to the inner ear: Kinetics and distribution. Hear. Res..

[28] Plontke S.K., Hartsock J.J., Gill R.M., Salt A.N. (2016). Intracochlear drug injections through the round window membrane: Measures to improve drug retention. Audiol. Neurotol..

[29] Talaei S., Schnee M.E., Aaron K.A., Ricci A.J. (2019). Dye tracking following posterior semicircular canal or round window membrane injections suggests a role for the cochlea aqueduct in modulating distribution. Front. Cell. Neurosci..

[30] Salt A.N., Hale S.A., Plonkte S.K. (2006). Perilymph sampling from the cochlear apex: A reliable method to obtain higher purity perilymph samples from scala tympani. J. Neurosci. Methods.

[31] Thorne M., Salt A.N., DeMott J.E., Henson M.M., Henson O., Gewalt S.L. (1999). Cochlear fluid space dimensions for six species derived from reconstructions of three-dimensional magnetic resonance images. Laryngoscope.

[32] Maxwell A.K., Hartl R.M.B., Greene N.T., Benichoux V., Mattingly J.K., Cass S.P., Tollin D.J. (2017). Semicircular canal pressure changes during high-intensity acoustic stimulation. Otol. Neurotol..

[33] Todt I., Mittmann P., Ernst A. (2016). Hearing preservation with a midscalar electrode comparison of a regular and steroid/pressure optimized surgical approach in patients with residual hearing. Otol. Neurotol..

[34] Carvalho G.J., Lalwani A.K. (1999). The effect of cochleostomy and intracochlear infusion on auditory brain stem response threshold in the guinea pig. Otol. Neurotol..

[35] Kim R., Riemann C., Kilgué A., Pfeiffer C., Scholtz L.-U., Sudhoff H., Todt I. Evaluation of intracochlear pressure during fluid application in the model and human petrous bone. Proceedings of the German Society of Oto-Rhino-Laryngology, Head and Neck Surgery.

